# Understanding the mechanisms of dormancy in an invasive alien Sycamore lace bug, *Corythucha ciliata* through transcript and metabolite profiling

**DOI:** 10.1038/s41598-017-02876-w

**Published:** 2017-06-01

**Authors:** Feng-Qi Li, Ning-Ning Fu, Cheng Qu, Ran Wang, Yi-Hua Xu, Chen Luo

**Affiliations:** Institute of Plant and Environment Protection, Beijing Academy of Agriculture and Forestry Sciences, Beijing, 100097 P. R. China

## Abstract

The sycamore lace bug, *Corythucha ciliata*, is a pest of sycamore trees. In China, it is found in the most northern border where it has been known to become dormant during harsh winters. But the molecular and metabolic basis for dormancy in this insect is still unknown. In this study, we analyzed the transcript and metabolite profiles of this bug to identify key genes and metabolites that are significantly regulated during dormancy in adult females and males. In total, 149 differentially expressed genes (DEGs) were significantly up-regulated and 337 DEGs were significantly down-regulated in dormant adults (both females and males). We found major differences in heat shock protein (HSPs), immunity-responsive genes, NAD-dependent deacetylase sirtuin-1 (SIRT1) and genes involved in the spliceosome pathway that is known to regulate stress. Among the 62 metabolites identified by GC-MS, 12 metabolites including glycerol, trehalose, and alanine were significantly increased during *C. ciliata* dormancy. By integrating the transcriptome and metabolite datasets, we found that the metabolites in glycolysis/gluconeogenesis and citrate cycle (TCA) were significantly reduced. This study is the first to report both transcript and metabolite profiles of the overwintering responses of *C. ciliata* to cold stress at the molecular level.

## Introduction

The sycamore lace bug, *Corythucha ciliata*, is an invasive exotic pest that is currently found worldwide. It is a native species of North America but eventually spreads to Europe, Asia and Africa^1–4^. It is known to cause massive destructions to sycamore trees and has huge ecological effects on the urban landscape. This insect overwinters as adults under the loose barks of sycamore trees and survives extremely low temperatures such as −30 °C^5^. Bugs breakout during spring in the southern cities of China including Changsha, Shanghai and Wuhan^3^.

Tolerance to low temperatures during winter has resulted in the expansion of *C. ciliata* into previously unaffected northern regions of China including Beijing, which is the northern most boundary of *C. ciliata* occurrence in China^6^, however, the physiological status (diapause or dormancy), molecular and physiological mechanisms during overwinter have not been characterized. In order to understand how this devastating insect pest adapted to extreme cold temperatures in winter, we investigated the ability of *C. ciliata* to develop cold tolerance in the winter temperatures and found the dormant status of this insect during overwinter. Furthermore, we present the first comprehensive transcripts and metabolite analysis of the mechanisms underlying cold tolerance of *C. ciliata* in winter. These results will assist in the development of successful management strategies for this species.

## Results

### Cold tolerance dormancy in *C. ciliata* adults

To determine the ability of *C. ciliata* to develop cold-tolerance in the winter temperatures, *C. ciliata* adults residing under the loose barks of *P*. *acerifolia* trees were collected from late September, 2015 to late February, 2016. We observed that in Beijing, the adults (female and male) overwintered under loose barks of trees from early November in 2015 (Fig. S1). The survival rate of adults was 91 ± 8% and 86 ± 11%, respectively on November 17 and November 26, 2015 and 50 ± 6% on January 21, 2016. When these inactive adults were maintained at room temperature they recovered their activity level. This finding suggested that *C. ciliata* adults were dormant and developed tolerance against severe cold stress in winter conditions.

**Figure 1:**
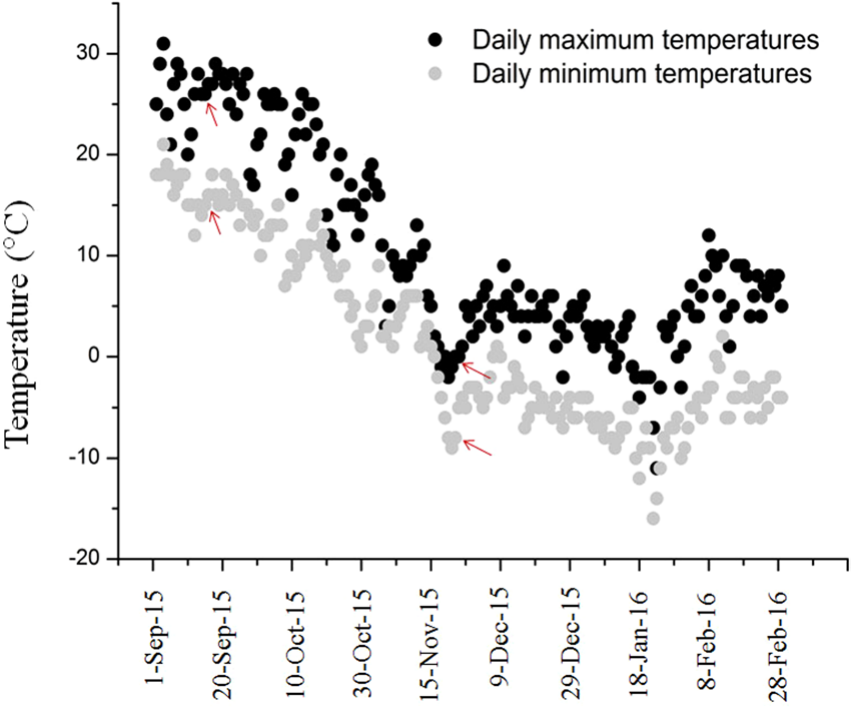
Daily maximum and minimum temperatures for Beijing city during September, 2015 to February, 2016. Red arrow Iindicates insect samples collection dates.

A comparative analysis between the transcript and metabolite profiles of the dormant adults in cold environments with the active adults at regular weather was conducted in this study. For this analysis, *C*. *ciliata* adults were collected on September 17 (active adults) and November 26 (dormant adults), 2015. As shown in Fig. 1, the maximum and minimum temperatures on September 17 and November 26 were 27/18 °C and 0/−8 °C, respectively. In addition, the samples collected on January 21, 2016 were also used to further verify the transcript by Quantitative Real-Time PCR (qRT-PCR). The maximum and minimum temperatures of that day was −26/−15 °C.

### RNA-seq of *C. ciliata* adults in dormant and non-dormant status

To reveal the cold tolerance-associated transcriptional responses in *C. ciliata*, RNA was extracted from four samples including active *C. ciliata* females and males (in temperatures from 18 °C to 27 °C) and dormant females and males (at winter temperatures from −8 °C to 0 °C) and were subjected to Illumina sequencing. The sequencing data from this study has been deposited in the NCBI Sequence Read Archive database under Accession numbers SRR3170922, SRR3170923, SRR3883369 and SRR3883370. High throughput sequencing generated 65,508,518–79,748,574 pairs of raw reads per sample and the Q20 scores (the average quality value) were more than 95% (Table 1). After filtering for quality, Trinity was used to assemble the 273,862,866 clean reads. The assembled reads from samples representing the different physiological stages (active and dormant stages) generated a total length of 61,678,837 bp and 92,218 unigenes. The mean length and N50 of these unigenes were 669 bp and 1,221 bp, respectively (Table 2). Analysis of the size distributions revealed that 13,342 unigenes were over 1,000 bp in length. All 92,218 *C. ciliata* unigenes were compared with the genes and proteins in seven public databases (Table 3) and the following results were obtained: BlastX yielded 22.4% (20,658 unigenes) that matched the NCBI-Nr database, BlastN resulted in 8.04% (7,421) of the unigenes that specifically matched the nucleotide sequences in the NCBI-Nt database, 18.04% (16,639) were similar to proteins in the Swiss-Prot database using BlastX, and finally 30.12% (27,785) were annotated in at least one database (Table 3). After CDS analysis by Blast and ESTscans^7^, 41,786 coding sequences were identified.

**Table 1:** Output statistics from Illumina sequencing 150 bp paired end reads of the active season versus dormancy adult life history stages of *C. ciliata*.

Sample	Raw Reads	Clean reads	Clean bases	Error(%)	Q20(%)	Q30(%)	GC(%)
NDM	67,466,456	65,036,134	8.13 G	0.03	95.16	90.51	45.93
NDF	79,748,574	76,903,406	9.61 G	0.03	95.24	90.67	44.87
DM	65,508,518	63,816,216	7.98 G	0.01	97.49	94.11	43.82
DF	70,023,910	68,107,110	8.51 G	0.01	97.58	94.31	43.84

NDM, Non-dormant male; NDF, Non-dormant female; DM, Dormant male; DF, Dormant female.

**Table 2:** Assembly statistics of *C. Ciliata* transcriptome.

	Min Length	Mean Length	Median Length	Max Length	N50	N90	Total Nucleotides
Transcripts	201	920	380	24465	2099	304	118156156
Unigenes	201	669	321	24465	1221	251	61678837

**Table 3:** Functional annotations of *C. ciliata* unigenes.

	Number of Unigenes	Percentage (%)
Annotated in NCBI-Nr	20,658	22.4
Annotated in NCBI-NT	7,421	8.04
Annotated in KO	8,698	9.43
Annotated in SwissProt	16,639	18.04
Annotated in PFAM	19,011	20.61
Annotated in GO	19,197	20.81
Annotated in KOG	11,775	12.76
Annotated in all Databases	3,043	3.29
Annotated in at least one Database	27,785	30.12

**Figure 2:**
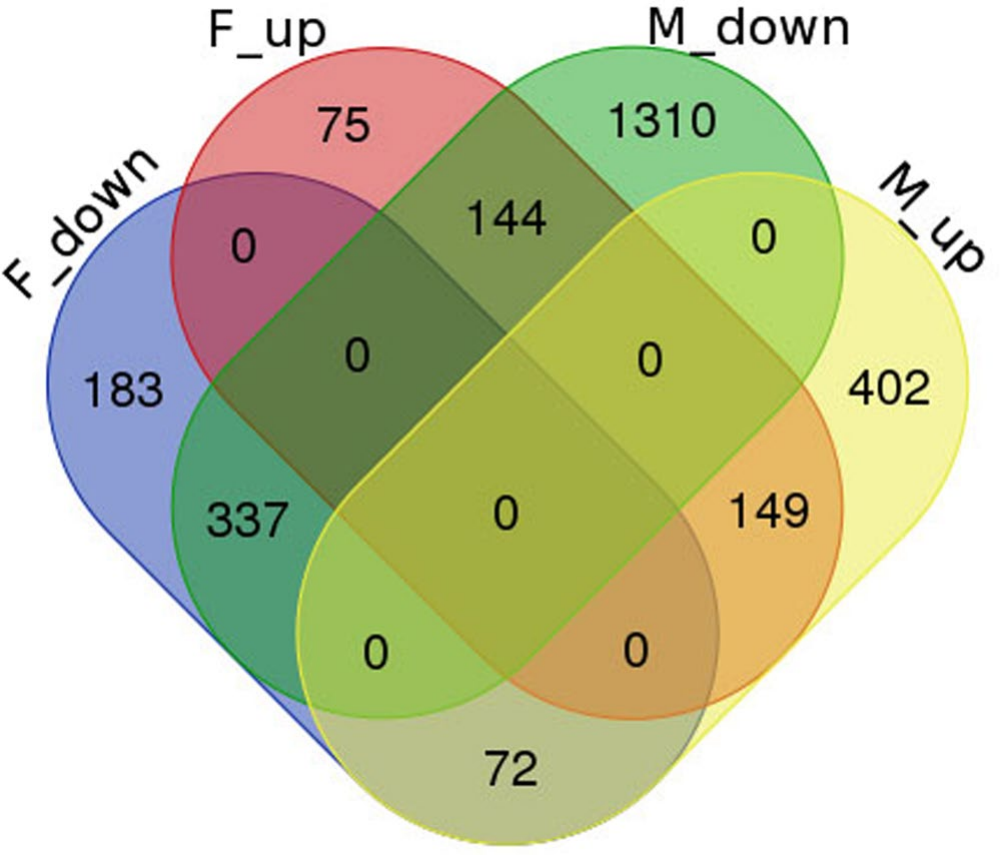
DEGs in adult male and female after dormancy. F_up: DEGs up-regulated in dormant female; F_down: DEGs down-regulated in dormant female; M_up: DGEsup-regulated in dormant male; M_down: DGEs down-regulated in dormant male.

To analyze the transcript abundance between the dormant and non-dormant samples, the RNA-seq reads from all four samples were mapped onto the assembled unigenes to determine DEGs (q-value < 0.005 and |log2 (foldchange)| >1). DEGs were defined as the transcripts that were significantly enriched or depleted in one sample relative to the other. As shown in Fig. 2, 623 and 1,791 DEGs were up- and down-regulated in a comparison between the dormant and non-dormant males, respectively. Moreover, 368 and 592 DEGs significantly differed between non-dormant and dormant females. More importantly, among the 486 DEGs that were differentially expressed in both adults (female and male), 149 were significantly up-regulated in both dormant adults (female and male) while 337 were significantly down-regulated in both dormant females and males (Fig. 2).

### Comparison of the metabolome of *C. ciliata* adults in dormant status

Comparative metabolomics analysis of dormant and non-dormant *C. ciliata* was conducted by GC-MS. A total of 62 metabolites were present in the samples, including 23 amino acids, 13 organic acids, 2 free fatty acids, 1 fatty acid methyl ester, 1 intermediate acidic metabolite, 5 sugars, 2 polyols, 3 phosphates, 2 ureides and 10 other metabolites. Interestingly, the metabolites quinic acid, L-cysteine, sucrose, fructose, glucose-1-phosphate, uridine, cytidine, cytidine monophosphate, tryptophan and arginine were not detected in the dormant adults but were identified in the non-dormant adults.

**Figure 3:**
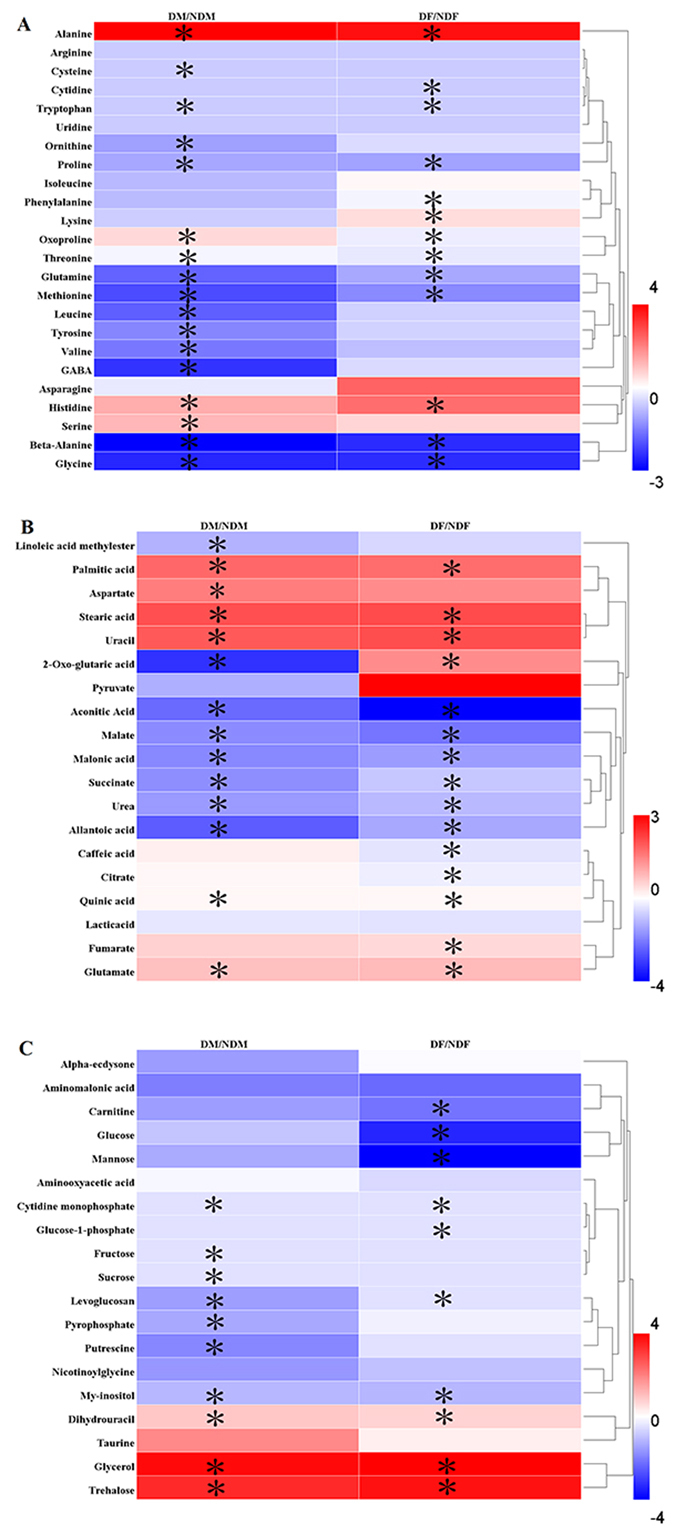
Comparison of the *C. ciliata* adults metabolome during dormancy. Heatmaps represent log2 fold change (dormant/non-dormant) of the A:amino acids, B:organic acids, C: sugars, polyols, and other metabolites of *C. ciliata*. Red and blue indicate up-regulation and down-regulation, respectively. DF: dormant female, NDF: non-dormant female, DM: dormant male, NDM: non-dormant male. Asterisks indicate significant differences (P < 0.05) between dormant and non-dormant state.

To analyze changes among the metabolites during *C. ciliata* dormancy, we determined the metabolic profile of *C. ciliata* before and after the onset of dormancy. Among the 62 metabolites identified in this study, 12 significantly increased, and 15 metabolites significantly decreased in response to dormancy status of both adults (female and male) (Fig. 3). The 12 increased metabolites included 2 free fatty acids (palmitic acid and stearic acid), 5 amino acids (alanine, histidine, oxoproline, serine and threonine), 1 sugar (trehalose), 1 polyol (glycerol), 1 organic acid (glutamic acid) and 2 other metabolites (uracil and dihydrouracil). Among them, trehalose was in a dominant proportion in the dormant samples, followed by alanine.

### Stress tolerance-related DEGs and SIRT1 were significantly up-regulated during dormancy

Among the 149 up-regulated DEGs, some were stress tolerance-related genes, including 6 HSP encoding genes (c52150_g1, c58999_g3, c48217_g1, c50355_g1, c54588_g1, c58999_g3), 1 defensin encoding gene (c38544_g1), and 1 leucine repeat-rich protein encoding gene (c55040_g1) (Supplementary Table S1). According to the phylogenetic analysis of HSP genes (Fig. S2), three (c50355_g1, c48217_g1, c52150_g1) belonged to HSP70 subclade, one (c57403_g1) belonged to HSP90 subclade, one (c58999_g3) belonged to heat shock cognate protein (HSC) subclade, and one (c54588_g1) was the ortholog of *Lygus Hesperus* HSP 21.4. Moreover, 2 HSP genes encoding HSP70 (c50355_g1 and c48217_g1) were the most up-regulated. Interestingly, 1 SIRT1 (c58006_g1) was also significantly up-regulated (Supplementary Table S1). The *C. ciliata* SIRT1 gene was very close to SIRT1 of *Zootermopsis nevadensis*, *Trachymyrmex zeteki*, *Camponotus floridanus* and *Melipona quadrifasciata* (Fig. S3). This gene acts upstream of the forkhead transcription factor (FOXO) in the FOXO signaling pathway^8^. It is likely that these stress tolerance-related DEGs and SIRT1 could be involved in inducing dormancy in both adult male and female *C. ciliata*.

### Spliceosome related genes were significantly up-regulated during dormancy

In the GO terms enrichment analysis, 24 GO terms were enriched and at least 14 of them were related to RNA splicing (Supplementary Table S2).

**Figure 4:**
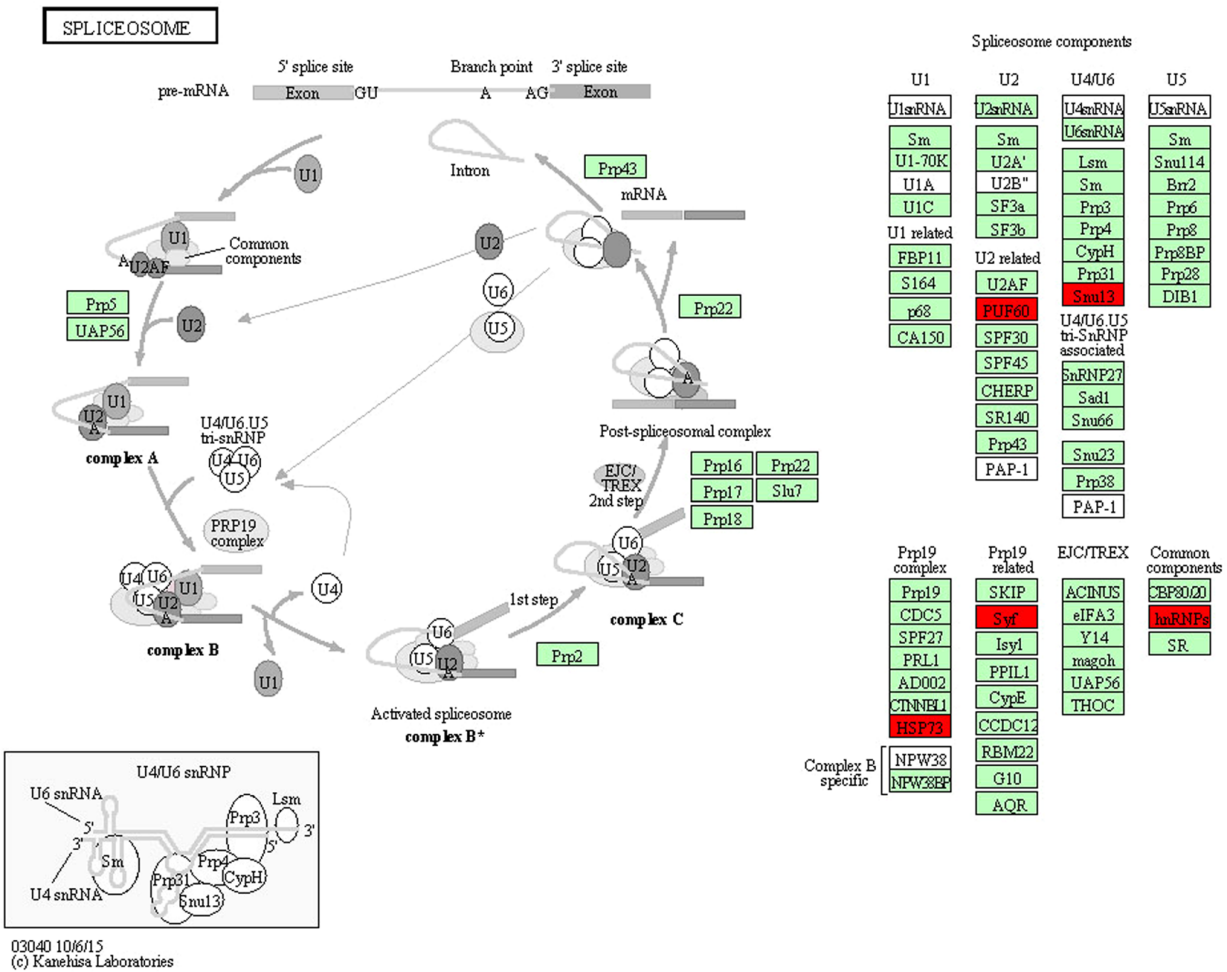
Cold-induced genes associated with the spliceosome pathway. 149 up-regulated DEGs were mapped to the KEGG pathway^36–38^ using KOBAS. Up-regulated genes are displayed in red.

In the KEGG enrichment analysis, the up-regulated DEGs were enriched only in the spliceosome pathway during dormancy (Supplementary Table S2). As show in Fig. 4, PUS60, Snu13 and others associated with U2, U4/U6, Prp19 as well as other common components were up-regulated during dormancy. In addition, HSP73 in the Prp19 complex, pre-mRNA-splicing factor SYF (SYF) in Prp19-related complex, and heterogeneous nuclear ribonucleoprotein (hnRNP) in common components were also up-regulated during dormancy (Fig. 4). This result indicated the importance of spliceosome in the establishment of cold tolerance in dormant *C. ciliata* female and male adults.

Among above genes, c30879_g1 encode NHP2-Like Protein (NHP2L/snu13), and *C. ciliata* NHP2L was close to NHP2L of *Tortanus dextrilobatus* and *Papilio xuthus* in phylogenetic analysis (Fig. S4). SYF (c64364_g1) is a component of the Prp19-related complex. Based on phylogenetic analysis of SYF gene (Fig. S5), *C. ciliata* SYF1 gene was very close to SYF1 of *Melipona quadrifasciata*, *Cyphomyrmex costatus*, *Eufriesea mexicana*, *Acromyrmex echinatior*, *Trachymyrmex zeteki*, and *Dufourea novaeangliae*. In addition, c61035_g1 was the ortholog of hnRNP in *Riptortus pedestris* (Fig. S6).

DEGs down-regulated by dormancy included 337 genes that were involved in the TCA cycle, carbon metabolism, valine, leucine and isoleucine degradation, fatty acid metabolism, pyruvate metabolism, and metabolic pathways (Supplementary Table S2). Since these pathways are related to metabolism, this is evidence for the well-known repression of basal metabolism during insect dormancy.

### Secondary metabolisms significantly depressed during dormancy

Among the 337 DEGs significantly down-regulated during dormancy, 74 were related to secondary metabolism. These DEGs as mentioned above were enriched in pathways such as TCA cycle and carbon metabolism (Supplementary Table S2). In contrast, up-regulated DEGs were not significantly enriched in any metabolism-related pathways during dormancy. This suggested a drastic repression of the secondary metabolism during dormancy than during the non-dormant status. Interestingly, 2 DEGs (c36229_g1 and c62170_g1) related to cytochrome P450 were up-regulated during the dormant status (Supplementary Table S1). Based on phylogenetic analysis (Fig. S7), c36229_g1 was the ortholog of *Apolygus lucorum* CYP6HM1, and c62170_g1 was the ortholog of *Nilaparvata lugens* CYP4CE1. This finding indicated that cytochrome P450 may play important role during the dormant status of *C. ciliata* adults.

**Figure 5:**
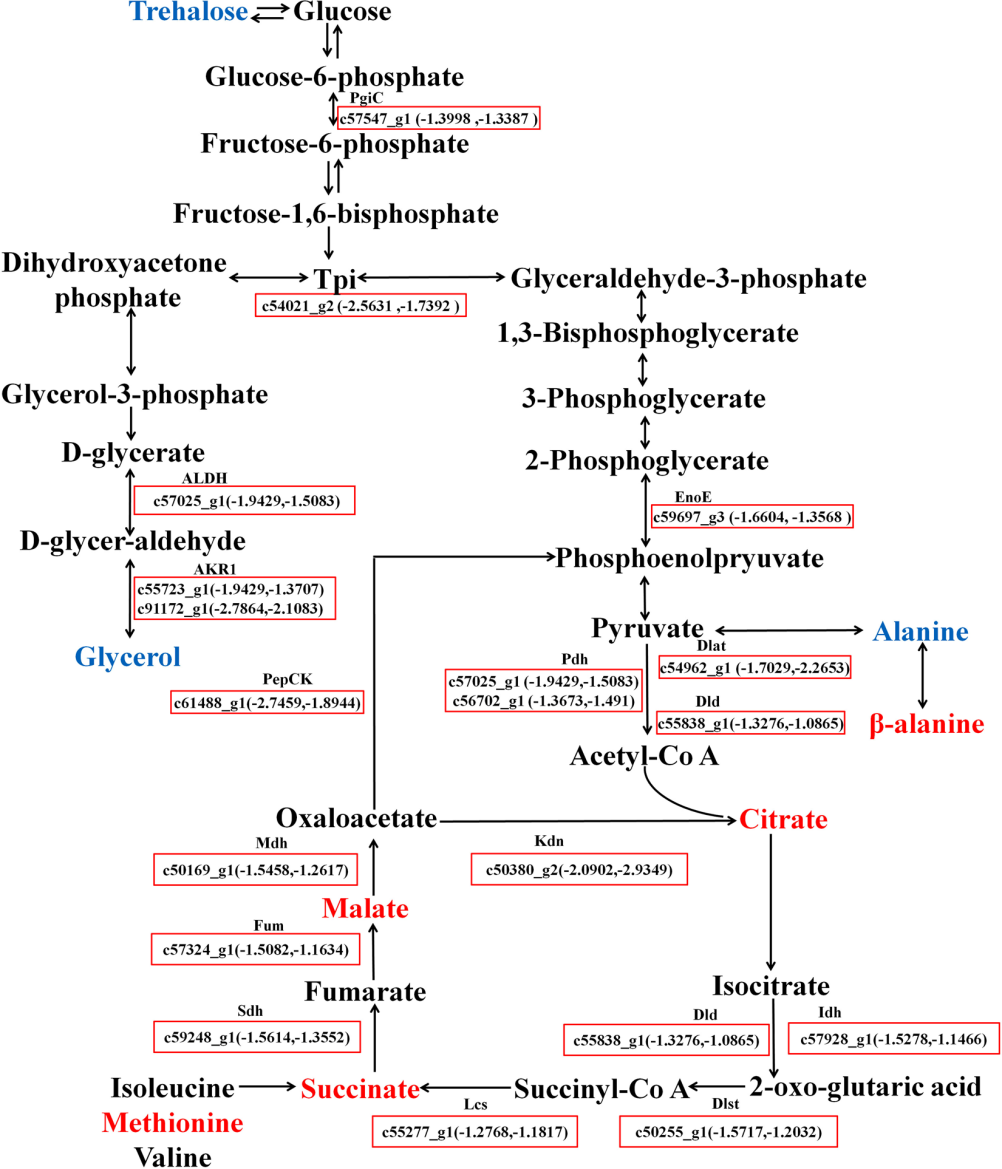
Genes involved in glycolysis/gluconeogenesis and TCA cycle were significantly repressed during dormancy. Names of enzymes are listed above the boxes, and the *C. ciliata* unigenes and log2 fold change values comparing dormant to non-dormant adults are listed within the boxes (First is the log2 fold change of dormant female vs non-dormant female, second is the log2 fold change of dormant male vs non-dormant male). Red boxes indicate DEGs that are significantly up-regulated in dormant compared with non-dormant adults with a q-value < 0.01. Metabolites in red text are significantly down-regulated in dormant adults relative to non-dormant adults. Metabolites in blue text are significantly up-regulated in dormant adults relative to non-dormant adults.

We then combined the RNA-seq and metabolomic datasets to construct the biological pathway diagrams for dormant *C. ciliata* (Fig. 5). Metabolites in glycolysis/gluconeogenesis and TCA cycle were repressed significantly (Fig. 5). This result indicated that in dormant *C. ciliata* adults substantial numbers of metabolites are suppressed than in non-dormant adults.

DEGs associated with cold tolerance included two enzymes, aldehyde reductase and aldehyde dehydrogenase, that are key enzymes promoted the glycerol production. These DEGs were also significantly down-regulated during dormancy compared with the non-dormant adults (Supplementary Table S1 and Fig. 2). In addition, 2 genes encoding trehalose-phosphate synthase were detected (c61898_g1 and c46175_g1). But, the expression of these genes did not change significantly between the dormant and non-dormant adults.

### qRT-PCR verification

**Figure 6:**
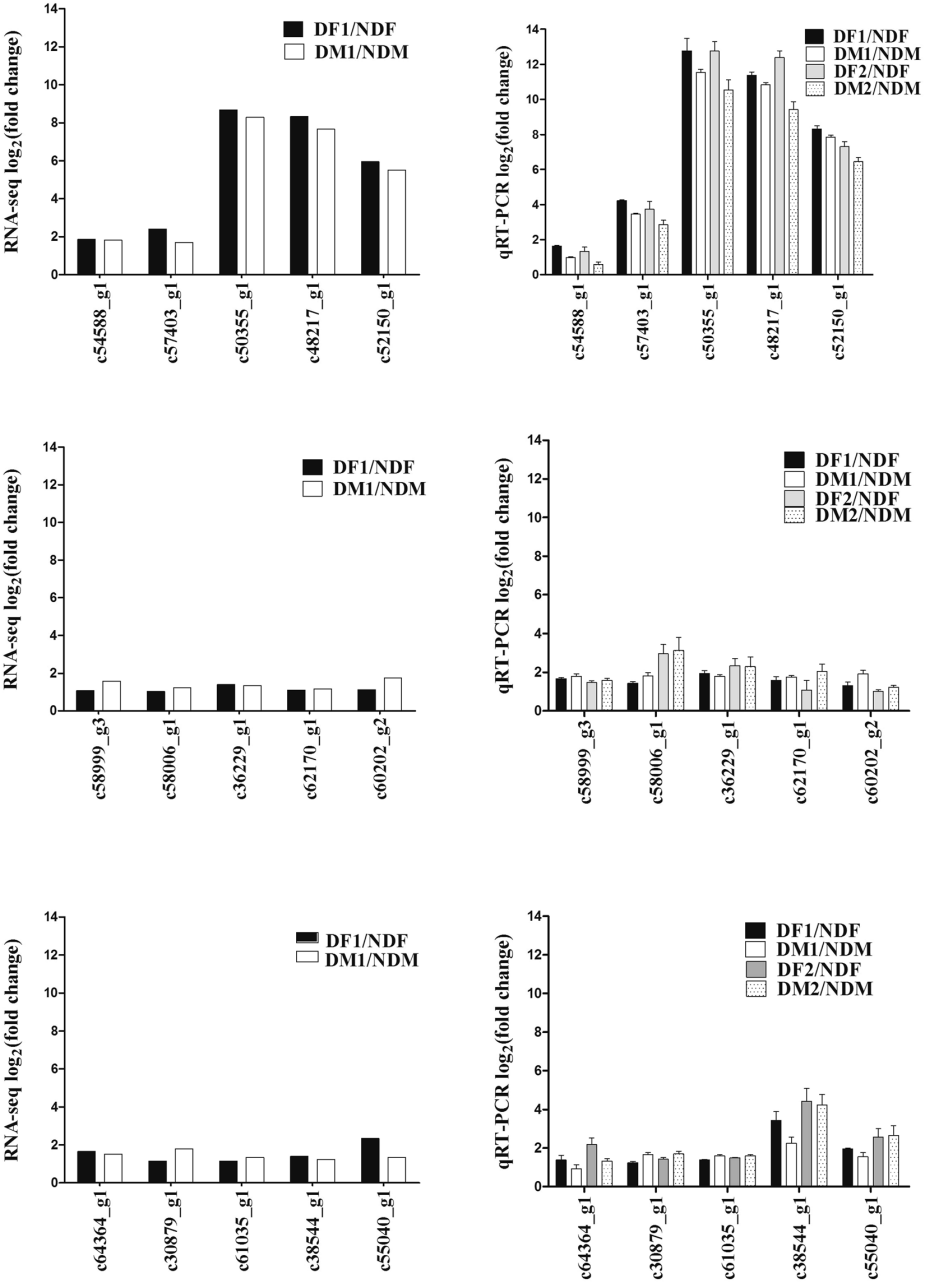
qRT-PCR validations of 15 DEGs during *C. ciliata* dormancy. The fold changes of 15 DEGs (dormant adults vs non-dormant adults) from RPKM values in RNA-Seq data and qRT-PCR data. DM1: dormant male collected in November 26, 2015(0 °C to −8 °C), DM2: dormant male collected in January 21, 2016 (−15 °C to −26 °C), NDM: non-dormant male, DF1: dormant female collected in November 26, 2015(0 °C to −8 °C), DF2: dormant female collected in January 21, 2016 (−15 °C to −26 °C), NDF: non-dormant female. The fold changes of the DEGs were calculated as the log2 value of each comparison from RNA-Seq and qRT-PCR data.

**Figure 7:**
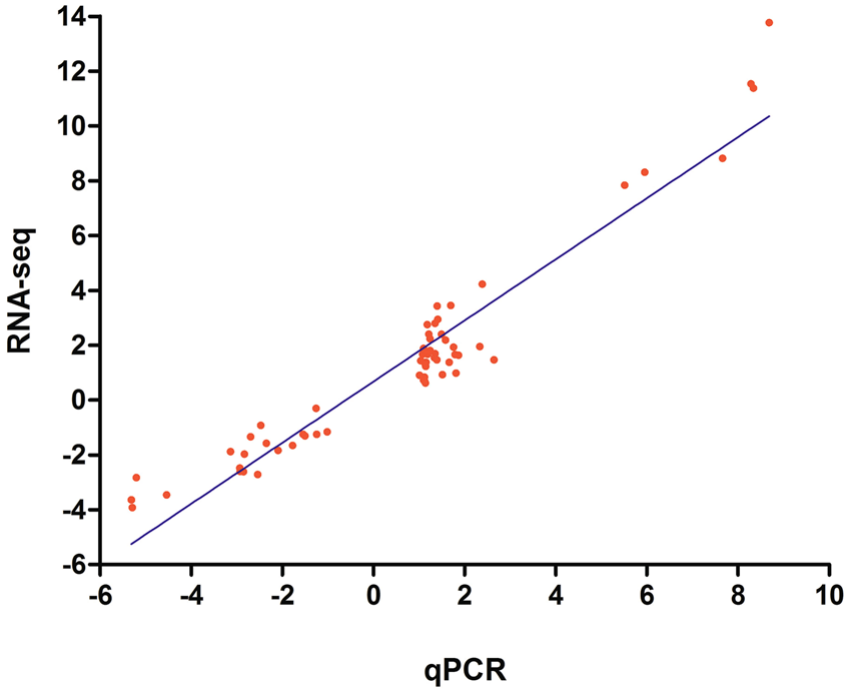
Coefficient analysis between gene expression fold changes obtained from RNA-seq and qRT-PCR data of 30 genes. The RNA-seq log2 values (fold changes; y-axis) were plotted against log2 value of qRT-PCR (x-axis).

To verify the reliability of transcript expression levels in our transcriptomic data, 30 DEGs (NDF vs DF or NDM vs DM) were tested by qRT-PCR (Figs 6 and 7). These include 15 described above genes and 15 randomly selected genes. These 15 described genes included 6 HSP genes (c54588_g1, c57403_g1,c50355_g1,c48217_g1,c52150_g1, c58999_g3), 1 SIRT gene (c58006_g1), 1 hnRNP gene (c61035_g1), 1 NHP2L gene (c30879_g1), 1 SYF gene (c64364_g1), 2 P450 genes (c36229_g1, c62170_g1), 2 immune and defense-related genes (c38544_g1, c55040_g1), 1 zinc finger CCHC-type and RNA-binding motif-containing protein 1 isoform X1 gene (c60202_g2). The sequences of these 15 genes have been deposited in the Genbank database (Supplementary Table S3). Other 15 randomly selected genes include 4 metabolism-related genes (c50169_g1, c50380_g2, c49134_g1 and c58522_g1), 1 neuropeptide GPCR A12 gene (c38999_g1), odorant binding protein 11 (c49990_g3), salivary secreted protein (c45793_g1), ATP synthase-beta (c48915_g1), 1 jnk interacting protein (c63313_g1), 1 obstructor B (c54508_g2), 1 CYCLE (c62280_g1), 1 predicted protein yellow-like (c56596_g1), 1 transcription factor Ken (c60889_g2), RNA-binding protein 34 (c58434_g1), and 1 predicted lysosomal-trafficking regulator (c65512_g1). The up-regulated expression levels of described 15 DEGs were verified by qRT-PCR (Fig. 6). The expression pattern of these DEGs was consistent with the data obtained from RNA-seq (Fig. 6), and the expression pattern of these 15 DEGs were stable between samples collected from November 26, 2015 and January 21, 2016. As shown in Fig. 7, the strong correlation between RNA-seq and qRT-PCR data was observed (Pearson’s r correlation coefficient = 0.918). Thus, the expression levels of genes observed in RNA-Seq in this study are reliable.

## Discussion

Dormancy is a common adaptation strategy in invertebrates to avoid extreme temperatures in the environment. Insects enter into dormancy to avoid the stresses of unfavorable seasons^9^. Overwinter of *C. ciliata* is achieved by dormancy that results in higher survival rate under harsh cold weather conditions in winter. This phenomenon may explain the rapid outbreaks of *C. ciliata* adults in the active season^10^.

This study reported the transcript and metabolite profiles of *C. ciliata* dormancy for the first time. We have identified novel genes and metabolites associated with cold tolerance in *C. ciliata*. We detected 149 significantly up-regulated DEGs in a comparison between non-dormant and dormant adults in both females and males. The variation in gene expression is not extensive, indicating that only a few key genes change their transcriptional activity during dormancy. Some of the identified genes related to overwintering in *C. ciliata* were similar to those previously reported for other insect species, such as HSPs and immune response genes^11^. Among them, HSP70 was the most up-regulated and in total 6 HSPs (3 HSP70, 1 HSP90, HSP21.4 and 1 HSC) were found to be uniquely expressed in dormant adults. This finding suggested the importance of HSPs during the dormancy of *C. ciliata*. It is well known that HSPs play a vital role in protein stabilization and/or cold tolerance in insect species as reported previously in *Drosophila melanogaster* ^12^, leafminer species^13^ and the mountain pine beetle, *Dendroctonus ponderosae* ^14^. In addition, several immune-response genes were also significantly up-regulated during dormancy, including defensin 4 and leucine repeat-rich protein. In *Drosophila*, HSP70 was also found to be related to immune response^15^. These DEGs may act to protect *C. ciliata* adults against pathogens when they undergo their dormant state under the loose barks of infested sycamore trees. Also, this finding indicated the crosstalk between cold tolerance and immune-defense responses.

Moreover, SIRT1 interacts with FOXO genes in the FOXO signaling pathway, which was reported to regulate overwintering diapause in the mosquito, *Culex pipiens* ^16^. However, further studies are needed to confirm the functions for these candidate DEGs in cold and immune tolerance pathways.

Interestingly, up-regulated DEGs were significantly enriched in the spliceosome pathway. Alternative splicing including exon skipping, mutually exclusive exons, alternative donor site, alternative acceptor site and intron retention events may result in one gene coding for multiple proteins. Previous studies indicated that alternative splicing is vital for the adaptative responses to multiple stress conditions^17–21^. Cold-induced genes have also been shown to play important roles in the spliceosome pathways in zebrafish^22^. To our knowledge, this is first report of the genes in the spliceosome pathway correlating to cold tolerance in insects except for *Drosophila* ^21^.

Furthermore, many DEGs that were significantly up-regulated during dormancy did not have functional annotations available in our databases. These new DEGs in *C. ciliata* adults provide a unique resource to understand insect dormancy. The characteristics of these genes will require functional studies in future.

Some known insect cryoprotectants including trehalose, alaine and glycerol have been reported to be up-regulated during dormancy^23^ consistent with our results in *C. ciliata* adults. Trehalose is vital for the stability of proteins and membranes during overwintering^24^ and is dominantly expressed in dormant samples suggesting its importance in *C. ciliata* overwintering. Glycerol is known to supercool the bodily fluids of insect, thus protecting them from lower temperatures^25^. In addition, the detected reduction in β-alanine possibly results from the conversion of L-alanine from β-alanine^26^. The down-regulation of aldehyde reductase, aldehyde dehydrogenase and trehalose-phosphate synthase could be attributed to their high concentrations and their constitutive expression during dormancy in *C. ciliata* adults^25, 26^.

Our transcript and metabolite datasets revealed the *C. ciliata* genes and metabolites that respond to cold stress environments in the northern most border of China. To fully understand the response of this invasive insect to different temperature stress, additional comparisons of transcript and metabolite levels should be made in future. In addition, to promote our understanding of the adaptation mechanism of Tingidae insect species to environmental stress, the transcript and metabolite information detected in this study can be compared to other Tingidae family species that experience environmental stress conditions in future.

## Methods

### Insect rearing and RNA isolation

Adult *C. ciliata* (both males and females) were collected from the *P*. × *acerifolia* trees cultivated in a garden maintained by the Plant Protection Institute, Chinese Academy of Agricultural Science in Beijing (40°01′24.13″, 116°16′46″). Insect collections were carried out in two seasons: the first was on September 17, 2015, which represents the regular (non-dormant) season and the second was on November 26, 2015, which represents the dormant season. In total there were four samples: males and females collected in the non-dormant and dormant seasons. Investigations of dormancy of *C. ciliata* were also conducted using the *C. ciliata* populations in this garden by observing the survival rate. Survival rate were calculated according to the formula: survival rate = A/T*100, where A = The active adults numbers of sycamore lace bug after maintained at room temperature (24–26 °C) one hour, and T = The total number of observed sycamore lace bug. One hundred individuals were tested in each times and repeat 4 times within each survey. Weather data for Beijing city was obtained from the China Meteorological Data Sharing Service System (http://cdc.cma.gov.cn).

Total RNA was extracted from pools of 500 individuals of each sample (males and females) using the RNAqueous-Micro kit (Life Technologies), and assessed using the Agilent Bioanalyzer 2100 system (Agilent Technologies, CA, USA).

### cDNA library preparation and Illumina sequencing

Illumina sequencing was performed on all four *C. ciliata* RNA samples representing the active males and females from the regular season (September 17, 2015), and the dormant males and females from the winter season (November 26, 2015). All RNA-seq libraries were prepared in accordance with the manufacturer’s protocol (Illumina Inc., San Diego, CA, USA) and 1.5 μg RNA each was used for library preparation, which was performed as described previously^27^. The libraries were then sequenced on an Illumina Hiseq 2500 platform (Illumina, San Diego, CA) to generate 150 bp paired-end reads.

### Bioinformatics analysis

From the raw sequenced reads the adapter sequences, reads containing poly-N and low quality reads were removed using in-house perl scripts. In addition, Q20, Q30, GC-content and sequence duplication levels in the clean data were also calculated. All downstream analyses were performed on the resulting clean data with high quality. Files on the left (read1 files) from all libraries/samples were pooled into one big left.fq file, and files on the right (read2 files) into one big right.fq file. These files were used for the transcriptome assembly using Trinity^28^ with min_kmer_cov set to 2 and all other parameters set to default. Unigenes were annotated based on the following seven databases: NCBI-Nr, NCBI-Nt, Pfam^29^, KOG/COG, Swiss-Prot, KO and Gene Ontology (GO)^30^ using BLAST with an E value threshold of 1e^−5^. For interesting genes associated with dormancy, the protein coding region of these genes were searched by ORF Finder (http://www.ncbi.nlm.nih.gov/projects/gorf/) and confirmed by PCR and sequence analysis. Phylogeny trees of were produced using neighbour-joining method with MEGA6.06^31^ and 1,000 replicates of bootstrap analyses.

### Differential expression analyses

Gene expression levels were estimated by RSEM^32^. Differential expression of genes between the active and dormant periods in the life cycle of *C. ciliata* was quantified using the DEGseq package^33^. P value was adjusted using q-value^34^ and unigenes that were expressed at significant levels (q-value < 0.005) and with more than 2-fold change (|log2FoldChange| >1) in expression level were considered to be differentially expressed. The GOseqR package was used to perform GO enrichment analysis of the DEGs, and the KOBAS software^35^ was used to perform the Kyoto Encyclopedia of Genes and Genomes (KEGG)^36–38^ pathway enrichment analysis.

### Metabolic Profiling

The four *C. ciliata* samples that were collected for RNA-seq analysis were also used for metabolic profiling. Whole *C. ciliata* bodies were snap-frozen in liquid nitrogen and stored at −80 °C until further use. Each of the four samples contained six biological replicates each with a pool of 200 adults. Samples were homogenized in 750 µl of cold (−20 °C) methanol/chloroform (2:1) using a glass homogenizer. Then, 500 µl ice-cold water was added to each sample and centrifuged at 2200 g for 15 min. About 150 µl of the resulting supernatant was transferred to a 1.5 ml tube and were dried using a vacuum concentrator. Then, we used a double derivatization method by first suspending the dried sample in 40 µl metoxyaminhydro chloride (Sigma-Aldrich, St. Louis, MO) and shaking for 2 h at 37 °C, followed by the addition of 70 μL N-methyl-N-(trimethylsilyl) trifluoroacetamide (Sigma-Aldrich) and another shaking for 30 min at 37 °C. Finally, we transferred an aliquot of each sample to a GC/MS glass vial for analysis by GC-MS (4D GC × GC-TOF-MS) (LECO, San Jose, USA).

The GC-MS system was equipped with a DB-5MS capillary column coated with 5% diphenyl cross-linked with 95% dimethylpolysiloxane (30 m × 250 μm inner diameter and 0.25 μm film thickness; J&W Scientific, Folsom, CA, USA). Then, 1 ml of each sample was injected at 230 °C in splitless mode with helium carrier gas flow set to 2 mL min^−1^. The initial temperature was maintained at 80 °C for 2 min, followed by a 15 °C per min ramp to 330 °C, and holding at this temperature for 6 min. Transfer line and ion source temperatures were 250 and 250 °C, respectively. Energy was set at −70 eV in an electron impact mode. Mass spectrometry data were acquired in a full-scan mode with an *m*/*z* range of 70 to 600 at a rate of 20 spectra per second after a solvent delay of 492 s. Chroma TOF 4.3X software (LECO Corporation) was used for data processing. Compounds were identified by searching against the LECO/Fiehn metabolite Mass spectral library and an in-house reference compound library. Concentrations were also corrected relative to an internal standard, arabinose, to adjust for any sample loss during extraction or injection. Metabolite contents were log2 transformed before analysis. Metabolite quantities were compared across the different treatments using ANOVA and Student’s *t*-test in IBM SPSS Statistics software 20. The metabolic and transcriptomic data were integrated by mapping the *Drosophila melanogaster* and *Acyrthosiphon pisum* biochemical pathways based on KEGG.

### qRT-PCR

cDNA was synthesized from all four samples using the PrimeScript RT Master Mix (Perfect Real Time) (TaKaRa) and used as template in qRT-PCR. The primers used in qRT-PCR are listed in Supplementary Table S3. The reactions were performed using the 2 × GoTaq ®qPCR Master Mix (Promega, Madison, WI, USA) and all reactions were conducted using the ABI Prism® 7500 (Applied Biosystems, Carlsbad, CA, USA) with the following PCR-cycling conditions: 95 °C for 2 min; followed by 40 cycles of 95 °C for 30 s, 60 °C for 1 min; then a final melting cycle at 95 °C for 15 s, 60 °C for 15 s, 95 °C for 15 s. Relative RNA expression was normalized to the level of alpha-tubulin. All samples had three biological replicates with three technical replicates. Relative expression levels of genes were quantified using the comparative 2^−ΔΔCt^ method^39^. The *C. ciliata* samples that were collected on September 17 (18 °C to 27 °C) and November 26, 2015 (−8 °C to 0 °C) were used for qRT-PCR. In addition, the samples collected on January 21, 2016 (−15 °C to −26 °C) were used to further verify the transcript by qRT-PCR.

## Electronic supplementary material

Supplementary figures

Supplementary Dataset 1

Supplementary Dataset 2

Supplementary Dataset 3
