# Supplementary material for: Understanding the mechanisms of dormancy in an invasive alien Sycamore lace bug, Corythucha ciliata through transcript and metabolite profiling
Source: Sci Rep. 2017 Jun 1;7:2631. doi: 10.1038/s41598-017-02876-w (PMC5453966; doi:10.1038/s41598-017-02876-w)
Supplement: Supplementary file 1 — Supplementary figures [file 41598_2017_2876_MOESM1_ESM.doc]

## Supporting Information

Article title: Understanding the mechanisms of dormancy in an invasive alien Sycamore lace bug, *Corythucha ciliata* through transcript and metabolite profiling

Authors: Feng-Qi Li, Ning-Ning Fu, Cheng Qu, Ran Wang, Yi-Hua Xu, Chen Luo

The following Supporting Information is available for this article:

Fig.S1.Images of *C. ciliata* in active and dormant periods. (A)In active periods, *C. ciliata* damaged on the leaves of sycamore trees.(B,C) In dormant periods, *C. ciliata* adults under the loose barks of sycamore trees.

Fig.S2. Phylogenetic tree of *C. ciliata* HSP genes and 16 HSP genes of other insect.

Fig.S3. Phylogenetic tree of *C. ciliata* SIRT-1 gene and 6 SIRT-1 genes of other insect.

Fig.S4. Phylogenetic tree of *C. ciliata* NHP2L gene and 8 NHP2L genes of other insect.

Fig.S5. Phylogenetic tree of *C. ciliata* SYF1 gene and 7 SYF1 genes of other insect.

Fig.S6. Phylogenetic tree of *C. ciliata* hnRNP gene and 6 hnRNP genes of other insect.

Fig.S7. Phylogenetic tree of *C. ciliata* P450 genes and 8 P450 genes of other insect.


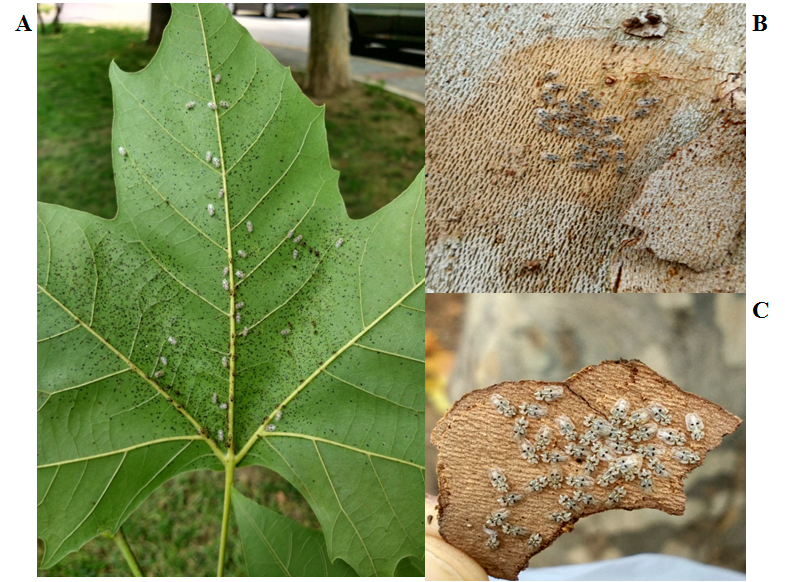


Fig.S1.Images of *C. ciliata* in active and dormant periods.

(A)In active periods, *C. ciliata* damaged on the leaves of sycamore trees.(B,C) In dormant periods, *C. ciliata* adults under the loose barks of sycamore trees.

Fig.S2. Phylogenetic tree of *C. ciliata* HSP genes and 16 HSP genes of other insect.

Fig.S3. Phylogenetic tree of *C. ciliata* SIRT-1 gene and 6 SIRT-1 genes of other insect.

Fig.S4. Phylogenetic tree of *C. ciliata* NHP2L gene and 8 NHP2L genes of other insect.

Fig.S5.Phylogenetic tree of *C. ciliata* SYF1 gene and 7 SYF1 genes of other insect.

Fig.S6. Phylogenetic tree of *C. ciliata* hnRNP gene and 6 hnRNP genes of other insect.

Fig.S7. Phylogenetic tree of *C. ciliata* P450 genes and 8 P450 genes of other insect.
